# Strategies Using Genomic Selection to Increase Genetic Gain in Breeding Programs for Wheat

**DOI:** 10.3389/fgene.2020.578123

**Published:** 2020-12-04

**Authors:** Biructawit Bekele Tessema, Huiming Liu, Anders Christian Sørensen, Jeppe Reitan Andersen, Just Jensen

**Affiliations:** ^1^Center for Quantitative Genetics and Genomics, Aarhus University, Tjele, Denmark; ^2^Nordic Seed A/S, Odder, Denmark

**Keywords:** wheat, genetic gain, genomic selection, stochastic simulation, genetic correlation

## Abstract

Conventional wheat-breeding programs involve crossing parental lines and subsequent selfing of the offspring for several generations to obtain inbred lines. Such a breeding program takes more than 8 years to develop a variety. Although wheat-breeding programs have been running for many years, genetic gain has been limited. However, the use of genomic information as selection criterion can increase selection accuracy and that would contribute to increased genetic gain. The main objective of this study was to quantify the increase in genetic gain by implementing genomic selection in traditional wheat-breeding programs. In addition, we investigated the effect of genetic correlation between different traits on genetic gain. A stochastic simulation was used to evaluate wheat-breeding programs that run simultaneously for 25 years with phenotypic or genomic selection. Genetic gain and genetic variance of wheat-breeding program based on phenotypes was compared to the one with genomic selection. Genetic gain from the wheat-breeding program based on genomic estimated breeding values (GEBVs) has tripled compared to phenotypic selection. Genomic selection is a promising strategy for improving genetic gain in wheat-breeding programs.

## Introduction

Conventional wheat-breeding programs use phenotypic values for selection of best individuals. Such programs are reported to yield yearly genetic gains that are lower than 1% (Godin et al., 2012). The procedure in phenotype-based selection involves creating genetic variation by crossing two parents followed by several rounds of selfing to create inbred lines. Resulting inbred lines are tested for a range of phenotypic parameters. Besides the most important trait, grain yield, breeders evaluate traits such as disease resistance, lodging, quality parameters, and a range of agronomical traits. In the early generations, breeders “visual preference” which is based on previous experience can also influence selection decisions. The aim of a breeding programs is to develop superior cultivars and the phenotypes of all the traits of interest are used for all selection decisions. However, it is the selection of individuals based on their breeding values, which would influence the response to selection in the next generation of a breeding cycle (Akdemir et al., 2019). In addition, selection of parental lines based on their breeding values will influence genetic gain in the subsequent breeding cycles. With the advent of molecular markers, selection decision was made by integrating information from both molecular markers and phenotypic data through marker-assisted selection (MAS) (Fujino et al., 2019). However, many complex traits including yield is under the control of many genes with small effects where MAS will be of a limited use due to low statistical power to detect individual genes (Bernardo, 2008). For many major crop plants, including wheat, many QTLs have been identified for many different traits, but the practical application of MAS faces many limitations (Bernardo, 2008) mainly because the QTL identified only account for a limited fraction of the genetic variance.

In contrast to MAS, genomic selection (GS) uses genome-wide markers to capture both large and small effect QTL to predict breeding values for complex traits (Meuwissen et al., 2001). Genomic prediction of expected breeding values will have advantages over phenotypic selection mainly because the accuracy in estimating breeding value is higher when genomic information is included for selection decision (Daetwyler et al., 2013). Genomic estimated breeding values (GEBVs) are calculated as the sum of effects related to genetic markers in linkage disequilibrium (LD) with one or more QTLs across the entire genome (Goddard and Hayes, 2007). Genomic selection uses a prediction model that is first trained using a population that contains both genotyped and phenotyped individuals. The trained model is then used to predict true breeding values of selection candidates. Such selection candidates may have no phenotypes and then their performance will be based on genomic information only. However, for selection candidates that are phenotyped and genotyped accuracy of prediction will improve due to optimal combination of genomic and phenotypic information.

A number of studies have explored application of genomic information in breeding programs for different plant species. Bernardo and Yu (2007) reported a 43% increase in genetic gain in a simulation study by integrating genomic information in maize breeding program compared to a program based on marker-assisted selection. A simulation study of Gaynor et al. (2017) compared wheat-breeding programs with and without genomic information and showed breeding program with genomic information outperformed phenotype based breeding program. A breeding program where selection decisions are only based on phenotypes aims to select best lines from a large segregating early generation and to evaluate fewer lines with greater replication in advanced generations. Integrating genomic selection in conventional wheat-breeding programs can increase genetic gain by selecting superior inbred line with higher selection accuracy (Bassi et al., 2015; Gaynor et al., 2017). In this way, only few changes are required in an already on-going phenotype-based wheat-breeding program. Generally, conventional wheat breeding starts by crossing selected inbred lines followed by several generations of selfing to get stable inbred lines for yield evaluations.

The motivation of this study comes from the observation that some of the simulation studies on genomic selection in wheat-breeding program does not mimic the complexity of actual wheat-breeding programs (Gaynor et al., 2017). In this simulation, we used real wheat genome data and the performance of genomic selection using real haplotype data would be closer to what happens in actual wheat-breeding program. Heritability of traits was based on real data study (Cericola et al., 2017). Plot size and density, yield plot at preliminary yield trial and advanced yield trial mimicked real wheat-breeding setup. In addition, the different actions taken at each breeding cycle stage reflected actual wheat-breeding programs. This was possible due to the use of the complex stochastic simulation program ADAM (Liu et al., 2019). The results of this study will contribute to the research on plant breeding as well as serve as a clear guideline for the plant breeding companies for implementation of genomic selection.

We applied genomic selection in conventional wheat-breeding programs to investigate the expected change in genetic gain. Stochastic simulation was used to quantify expected genetic gain in conventional wheat-breeding programs when incorporating genomic information in making selection decision. Stochastic simulation allow to model breeding schemes that mimics an actual breeding program on a very detailed level (Liu et al., 2019). For instance, stochastic simulation can be used to simulate an entire population of individual plants and it can accounts for the change in allele frequencies caused by selection. It also allows simulation of many rounds of selection and results in a more accurate estimate of genetic variance and response to selection. This makes stochastic simulation very precise in predicting consequences of alternative breeding schemes. Studies have also showed the benefit of stochastic simulation (Wensch-Dorendorf et al., 2011; Gaynor et al., 2017).

In the present simulation work, we compared phenotype-based selection programs with genomic-based selection programs, where both selection criteria applied in conventional wheat-breeding programs. To achieve this, phenotype and genomic-based breeding schemes were simulated following a standard commercial wheat-breeding program, where the number of crosses, families, and single plants mimic real life wheat-breeding program. The aims of this study are to (1) investigate if incorporation of genomic information in wheat breeding program increases genetic gain, (2) study the change in genetic variance between phenotypic and genomic selection, and (3) investigate accuracy of selection in phenotypic and genomic selection.

## Materials and Methods

### Simulation Design

A stochastic simulation model was used to simulate a conventional wheat breeding strategy that run for 25 breeding cycles. Simulations were carried out using ADAM software (Liu et al., 2019). Two different wheat breeding strategies were simulated, (1) phenotypic selection and (2) genomic selection. Phenotypes and underlying genotypes were simulated including both QTL and markers. Based on phenotypic (and marker) information (G)EBV were predicted using a linear model.

**FIGURE 1:**
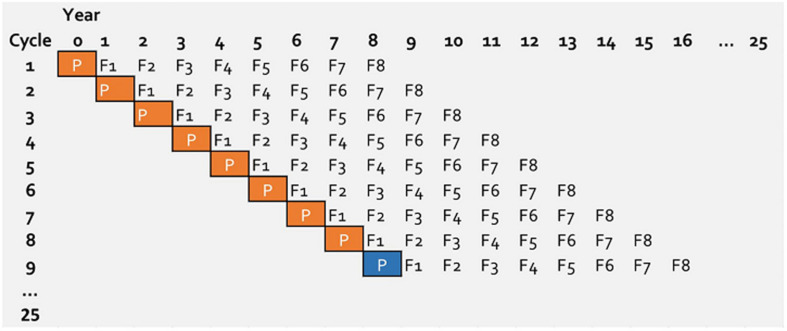
Structure of simulated wheat breeding program running over 25 years. Every year a new breeding cycle is initiated. For the first 7 years parents (P with orange box) are selected from the base population and after year 7 parents (P with blue box) is selected from previous cycles. For genomic selection the first 8 years are burn-in period.

A new breeding cycle was initiated every year and the breeding cycles, therefore, were overlapping (Figure 1). A breeding cycle represent eight generations from initial crossing until final elite line selection. A breeding cycle starts with parental lines (P), followed by generation F1 up to F8, where the number represent the generation in which they are generated (Figure 1). Since the breeding cycles are overlapping, in a given year, events (selection or mating) on eight different cycles were simulated. This allowed movement of information between cycles every year.

In a practical genomic wheat-breeding program, genomic selection would be implemented in an on-going phenotypic selection program. To mimic this, from the simulated 25 years of breeding program for genomic selection, the selection in the first 8 years was based on phenotypes. This means genomic information was introduced on the ninth year of the breeding program. This burn-in stage has also served to create differences between breeding cycles.

### Simulation of Founder Population

The genome for the founder population was generated from a realized wheat genome data set (Cericola et al., 2017) that were read into ADAM software. The genome data set represents commercial wheat lines obtained from three breeding cycles and includes 988 F6 lines. In the first generation of founder population, all SNPs are evenly distributed across the total (21) chromosomes and every *N*th locus harbors a QTL that code for a trait under selection and the remaining loci are genetic markers. Thus from the total 9582 markers, 1039 loci were randomly chosen across the genome was assumed as QTLs while the remaining 8543 loci were assumed as anonymous markers.

The extent of linkage disequilibrium in the genome data set is explained in Cericola et al. (2017). The average *r*^2^ within chromosomes was 0.05, indicating the presence of low LD decay. The average *r*^2^ for the genome A, B, and D was 0.05, 0.05, and 0.11, respectively. The average distance of markers with *r*^2^ > 0.5 was 9.47, 8.38, and 7.73 cM for genome A, B, and D, respectively.

### Simulation of Base Population

A base population of 480 lines were generated from the founder population. The genotype of each line was sampled from a pool of chromosomes of the founder population. Each line was generated by, for each chromosome 1 to 21, randomly sampling one chromosome without replacement from the pool of chromosomes. Then the second chromosome is set to be identical to the first one to generate a fully inbred line.

Parental lines were chosen from the base population for the first seven breeding cycles. The breeding program was run in parallel, meaning that a new breeding cycle was started every year. After this stage, parents were selected randomly from F6, F7, and F8 of the previous breeding cycles.

### Simulation of Phenotypes

Three traits for selections were simulated and these were breeder’s visual preference (BVP), yield at preliminary yield trial (PYT) and yield at advanced yield trial (AYT). The observation of the traits were realized at the different stages of a breeding cycle, breeder’s visual preference (BVP) at F2 and F4 generation, yield at preliminary yield trial (PYT) at F5 and yield at advanced yield trial (AYT) at F6 and F7 generations. AYT was also applied for family selection at F3. The preliminary yield trial represents un-replicated plot with limited amount of seeds sown sparsely. Whereas advanced yield trial is a standard yield plot with normal seed density and plots replicated three times in three different locations. The phenotype, *y*, was calculated as, *y* = *g* + *e*, where *g* is the true additive-genetic value and *e* is residual value. True breeding values (TBVs) of the traits were determined by summing the allelic effects of its QTL. The QTL effects $\left(a_{j}^{\prime }\right)$ was sampled from multivariate normal distribution, that is aj=aji×σq⁢t⁢l2∑kn2⁢p⁢k⁢(1-p⁢k)⁢ak2′, where subscripts *k* and *j* denote the QTL *k* and QTL *j*, *p*_*k*_ and *p*_*j*_ are the minor allele frequencies of QTL *k* and QTL *j*, $a_{k}^{\prime }$ and $a_{j}^{\prime }$ are the substitution effect of QTL *k* and QTL *j* before being scaled. When simulating correlation between traits, additive genetic variance and heritability of each trait was specified along with the genetic and residual correlations between traits. The additive genetic covariance matrix was then derived from the additive genetic variances and the additive genetic correlation matrix (Faux et al., 2016). The genetic variance for each trait was set to 1 (standardized unit) in the base population. The residual effect (e) for each individual is sampled from a normal distribution $N∼\left(O,\sigma_{e}^{2}\right)$. The residual was simulated independently assuming phenotypic correlation from genetic correlation. Heritability (h^2^) for BVP was set 0.1 and calculated $h^{2}=\frac{\sigma_{g}^{2}}{\sigma_{g}^{2}+\sigma_{e}^{2}}$. Plot heritability $\left(h_{p\,l\,o\,t}^{2}\right)$ for yield at PYT and AYT were 0.2 and 0.3, respectively, based on results of Cericola et al. (2017) which was based on results from a large commercial wheat population. In order to achieve targeted plot heritability for yield trait, the value for residual variance $\left(\sigma_{e}^{2}\right)$ was calibrated following this equation derived from Falconer and Mackay (1996) under the assumption of Hardy-Weinberg Equilibrium.

$$\sigma_{e}^{2}=\frac{2\,N_{p}\,H\,\sigma_{g}^{2}\,\left(1-h_{p\,l\,o\,t}^{2}\right)}{h_{p\,l\,o\,t}^{2}}-\left(1-H\right)\,\sigma_{g}^{2}$$

Where H is expected frequency of homozygosity in the generation, $\sigma_{g}^{2}$ is genetic variance, $\sigma_{e}^{2}$ is residual variance.

### Statistical Model

Breeding value estimation was done using multi-trait BLUP model in DMU software (Madsen and Jensen, 2013). For genomic selection a GBLUP model was implemented.

$$\left[\begin{array}{c} y_{1}\\ y_{2}\\ y_{3} \end{array}\right]=\left[\begin{array}{ccc} 1_{1} & 0 & 0\\ 0 & 1_{2} & 0\\ 0 & 0 & 1_{3} \end{array}\right]\,\left[\begin{array}{c} \mu_{1}\\ \mu_{2}\\ \mu_{3} \end{array}\right]+\left[\begin{array}{ccc} Z_{1} & 0 & 0\\ 0 & Z_{2} & 0\\ 0 & 0 & Z_{3} \end{array}\right]\,\left[\begin{array}{c} a_{1}\\ a_{2}\\ a_{3} \end{array}\right]+\left[\begin{array}{c} e_{1}\\ e_{2}\\ e_{3} \end{array}\right]$$

Where $\left[\begin{array}{c} y_{1}\\ y_{2}\\ y_{3} \end{array}\right]$ is the vector of traits BVP, PYT and AYT, and **1_1,_ 1_2_ and 1_3_** are the identity matrices, $\left[\begin{array}{c} {µ}_{1}\\ {µ}_{1}\\ {µ}_{1} \end{array}\right]$ is the vector of population means of BVP, PYT and AYT, $\left[\begin{array}{c} a_{1}\\ a_{2}\\ a_{3} \end{array}\right]$ is the vector of additive genetic effects of the three traits. ***Z*_1_, *Z*_2_** and ***Z*_3_** are the design matrices that associate breeding values with BVP, PYT and AYT and $\left[\begin{array}{c} e_{1}\\ e_{2}\\ e_{3} \end{array}\right]$ is the vector of residual errors of BVP, yield at PYT and AYT. It is assumed that $\left[\begin{array}{c} a_{1}\\ a_{2}\\ a_{3} \end{array}\right]$∼*N*(O,*G*⊗**G**_0_) where ***G_O_*** is $\left[\begin{array}{ccc} \sigma_{a\,1}^{2} & \sigma_{a\,12} & \sigma_{a\,13}\\ \sigma_{a\,21} & \sigma_{a\,2}^{2} & \sigma_{a\,23}\\ \sigma_{a\,21} & \sigma_{a\,32} & \sigma_{a\,3}^{2} \end{array}\right]$ when genomic information is used and $\left[\begin{array}{c} p_{1}\\ p_{2}\\ p_{3} \end{array}\right]$∼*N*(O,*I*⊗**I**_o_) where $I_{0}\,\left[\begin{array}{ccc} \sigma_{p\,1}^{2} & \sigma_{p\,12} & \sigma_{p\,13}\\ \sigma_{p\,21} & \sigma_{p\,2}^{2} & \sigma_{p\,23}\\ \sigma_{p\,31} & \sigma_{p\,32} & \sigma_{p\,3}^{2} \end{array}\right]$ when phenotypic information is used. $\left[\begin{array}{c} e_{1}\\ e_{2}\\ e_{3} \end{array}\right]$∼*N*(O,*I*⊗**R**), where ***R*** is the residual variance covariance matrix of the three traits. The three traits are measured on different lines and different years meaning the residual variance is independent and have value of zero.

### Breeding Schemes

#### Phenotypic Selection (PS) Breeding Scheme

A breeding program running for 25 years was simulated following a conventional winter wheat breeding structure (Figure 1). Every year in the breeding program a new breeding cycle starts.

**FIGURE 2:**
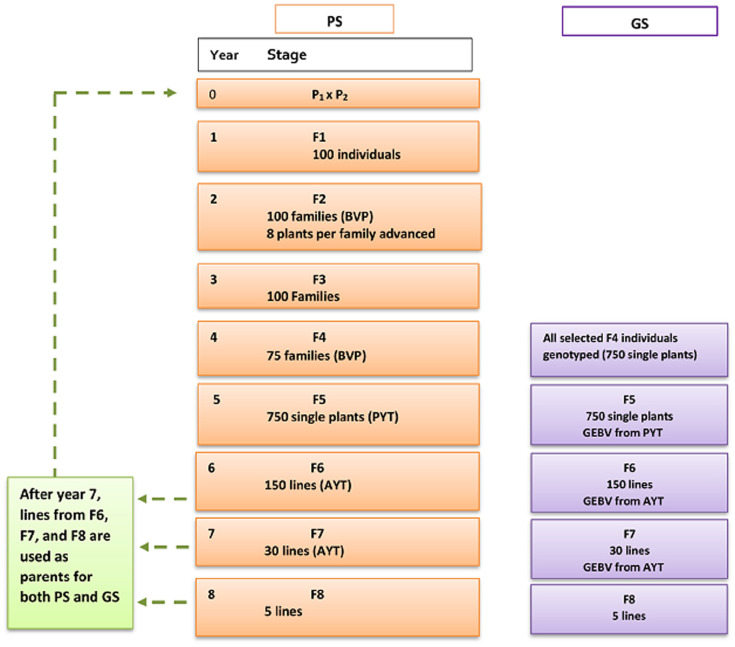
Breeding scheme for conventional phenotypic selection (PS) and genomic selection (GS) of wheat-breeding program. PS and GS breeding scheme has similar setup until F4. For GS, GEBV was used for selection starting from F5. BVP = Breeders visual preference, PYT = preliminary yield trial, AYT = advanced yield trial, GEBV = genomic estimated breeding value.

**Parents (P):** For the first seven breeding cycles, 60 parental lines were chosen randomly from the base population while for the rest of 18 breeding cycles parents were selected based on their breeding values from F6, F7, and F8 of the previous breeding cycles and this allowed elite lines to be used for crossing (Figure 2). Each parental line was used for a maximum of six crosses and from the total 1770 possible crosses, 100 crosses were randomly chosen.

**F1:** One hundred F1 plants were generated and each F1 was allowed to produce 30 (F2) seeds. Families from F2 to F4 share a common ancestor (F1), thus families from F2 to F4 were referred as F1 families.

**F2**: In total, there were 100 plots and each F2 plot had 30 plants. With-in each F1 family eight highest-ranking single plants were selected based on their breeding value for trait 1 (BVP). Heritability of 0.1 was assigned for the trait BVP. Selected eight single plants from each family were advanced to the next stage in the breeding program.

**F3**: Each F1 family was planted in three plots and evaluated based on yield performance. The plot set up was similar as in AYT. Out of the 100 families, 75 highest ranking families based on their breeding values were advanced to the next stage.

**F4**: The advanced 75 F1 families were planted in un-replicated field trials. Ten single plants per family were selected based of BVP in order to form individual lines based on single seed descent. Selected 750 lines were advanced to preliminary yield trial (PYT).

**F5**: F5 lines were created by advancing selected F4 individuals through single seed descent (SSD). A single plot was simulated to generate 50 plants that were genetically identical. This preliminary yield trial (PYT) is un-replicated and this step is mainly done for the purpose of seed multiplication. Yield recording was made on each plot. Plot mean was used to select 150 best lines from the available 750 lines. Selected lines were stored to be potentially used as parents in the proceeding breeding cycles.

**F6**: The 150 lines selected from F5 were evaluated in advanced yield trail (AYT), where plots and trials were replicated three times across three different locations. However, yield evaluation here was made based on the assumption that there is no genotype by environment interaction and heritability is chosen to reflect the part of additive genetic variance that is common to different environments (Cericola et al., 2017). The AYT trait was set as a selection criteria. Thirty best lines were selected from the 150 lines to be advanced to the next stage. The selected lines will be added to a pool of potential parents.

**F7**: The selected 30 best lines were evaluated for yield and the field trial setup was similar to the AYT in F6. Five best lines are selected based on yield and were selfed to produce F8. Besides, the selected lines will be stored to potentially become parents. The best F8 lines are sent to an official yield trial test to be released as varieties.

### Genomic Selection (GS) Breeding Scheme

The proposed genomic wheat-breeding program was designed to introduce genomic selection starting from the preliminary yield trial stage, F5 (Figure 2). The genomic breeding program combines genotype and phenotype information to predict GEBVs for yield. The first 8 years of the breeding program was a burn-in phase which had similar set up as phenotypic selection described earlier, which means genomic information was introduced in the breeding program at year 9. The genomic breeding program from year 9 to 25 is described as follows.

**F4:** For genomic selection breeding strategy, all selected 750 single plants were genotyped in each cycle. F4 genotypes together with phenotypes for the targeted traits were added yearly for building the reference population. The initial reference population at year 16 (cycle 16) contained 750 genotypes. Every year the reference populations increased by 750 new genotypes.

**F5**: Each F5 plot had 50 plants descended from the single seed and only one replicate was simulated. Yield was recorded on all F5 plots in each F4-line. Line selection was done based on plot yield performance of 750 F4-lines. The phenotypes and genotypes of 750 F4-lines together with the existing reference population were used to estimate breeding values of each lines using GBLUP model (Madsen and Jensen, 2013). Thus, GEBVs of PYT were used to select the highest ranking 150 lines to be advanced to the next stage. The information of phenotypes and genotypes in the current breeding cycle was stored to be used for predicting breeding values in the next cycles. The germplasm of the 150 selected lines were stored and potentially become parental lines for the proceeding cycles.

**F6**: For each F6 line, nine replicates of plot was simulated. 150 F6 lines are phenotyped for yield for all the nine replicates in an advanced yield trial. Each F6 plot had 1500 plants. The phenotype of 150 lines combined with the genotype information of their corresponding F4 genotypes were used for prediction of breeding values. Based on GEBV of AYT, the 30 highest-ranking lines were selected from 150 lines. The selected lines were stored to be used as parents in the proceeding breeding cycles.

**F7**: The advanced 30 lines were evaluated for yield on all the nine replicates similar to F6. Each plot had 1500 plants. The average yield performance of each plot was recorded. The phenotype and genotype of the 30 lines together with the existing reference population was used to estimate breeding values. Five lines out of 30 lines were selected based on their GEBVs.

For both GS and PS, five scenarios were simulated with five different levels of genetic correlation between PYT and AYT. The correlation levels are 0.1, 0.3, 0.5, 0.7, and 0.9. The correlation of BVP with PYT and AYT was 0.1 for all the five scenarios. Each scenario in the simulation was replicated 10 times.

### Additive Genetic Variance

Additive genetic variance was computed in generation F5 as the variance of mean TBVs of all the individuals in each breeding cycle. Generation F5 is the breeding cycle stage where there is highest selection intensity.

### Prediction Accuracy

Selection accuracy for phenotypic selection and genomic selection was evaluated as a correlation between predicted breeding values and TBVs in each cycle. For phenotypic selection, accuracy was calculated as a correlation between plot phenotype and TBVs while for genomic selection it was a correlation between plot GEBVs and TBV.

### Comparison of Breeding Strategies

The expected genetic gain was quantified from each simulated breeding strategy. Genetic gain comparison on F8 generation was done by plotting the mean of true breeding values (TBVs) of F8 individuals against time. For the comparison of conventional and genomic breeding strategies, data from years 9 to 25 was used in the analysis. The annual genetic gain was computed for each scenario by regressing TBVs of AYT on time with the assumption of linear response from years 9 to 25 within 10 replicates. The standard deviation of genetic response for AYT is estimated as a measure of uncertainty of the breeding program. The development of genetic variance over several rounds of selection was compared between genomic and phenotypic selection. Genetic variance per breeding cycle (F1–F8) is plotted over time. The change in genetic variance for all scenarios at the end of breeding cycle is presented. Plotting and calculation was done using R statistical programming language and environment (R Core Team, 2019).

## Results

### Genetic Gain

**FIGURE 3:**
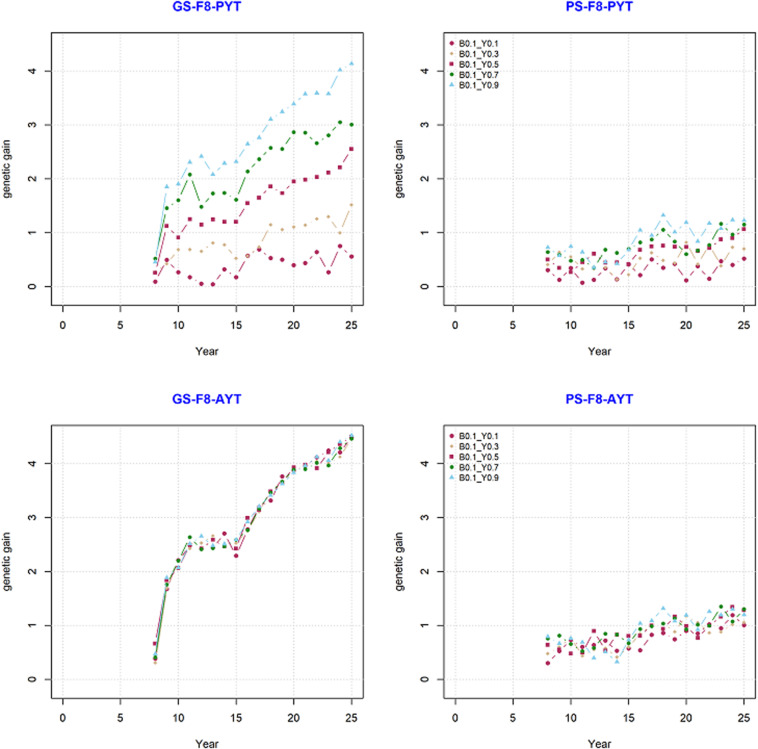
Genetic gain for the final product (F8) for PYT and AYT for genomic and phenotypic selection. The five levels of correlation (Y0.1, Y0.3, Y0.5, Y0.7, and Y0.9) between PYT and AYT, and correlation of BVP with PYT and AYT (B0.1) is shown.

Genetic gain from phenotypic selection and genomic selection was compared based on their mean breeding values over the period of 25 years. The comparison was done for trait PYT and AYT of the F8 generations since they were the end product in the current simulation. Our simulation result showed that the breeding program that uses genomic information generated significantly higher genetic gain than the breeding program with only phenotypic selection (Figure 3). The change in genetic gain when GS is introduced at year nine to an already ongoing PS can be clearly seen in Figure 3. There is a high jump in genetic gain at year nine in GS compared to PS. The trend in genetic gain was increasing at higher rate over time in GS compared to PS. The difference in genetic trends can also be seen among different level of genetic correlations between PYT and AYT within both PS and GS (Figure 3).

Genetic gain within PS and GS was also compared based on the level of genetic correlation between PYT and AYT and there were five level of genetic correlations (0.1, 0.3, 0.5, 0.7, and 0.9). According to the level of genetic correlations between traits there were a difference in mean breeding value with-in each selection criteria (PS and GS). The difference in genetic gain for the different levels of correlation can be clearly seen for PYT than AYT. The reason for this is that AYT was considered as the breeding goal and thus economic weight was assigned to AYT. This means genetic responses for PYT depends on the level of correlation with AYT for GS, the higher the correlation with AYT the higher genetic gain for PYT. This happened because of the genetic correlation between PYT and AYT as well as the genomic information available from the previous cycles are used for prediction. In addition, selection intensity at PYT is higher than AYT. However, the highest genetic gain is realized for AYT. The different levels of genetic correlation for AYT did not show significant differences among the different correlations. The standard deviation (SD) of the different scenarios for PS and GS breeding schemes tells the uncertainty of the breeding program. The SD of the individual estimates for AYT ranged 0.310–0.378 for PS whereas for GS the range is 1.127–1.143 (Table 1).

**TABLE 1:** Rate of genetic gain (Δ*G*) for AYT in genetic standard deviation with standard errors in brackets from year 9 to 25 and for 10 replicates and standard deviation (SD) of AYT for PS (phenotypic selection) and GS (genomic selection).

	**Δ*G***		**SD**	
**Scenario**	**PS**	**GS**	**GS**	**PS**
B0.1_Y0.1	0.035(0.001)	0.186(0.004)	1.141	0.310
B0.1_Y0.3	0.041(0.001)	0.185(0.002)	1.138	0.345
B0.1_Y0.5	0.044(0.001)	0.186(0.002)	1.143	0.356
B0.1_Y0.7	0.045(0.001)	0.183(0.002)	1.127	0.348
B0.1_Y0.9	0.049(0.001)	0.183(0.003)	1.127	0.378

*The five level of correlation (Y0.1, Y0.3, Y0.5, Y0.7, and Y0.9) between PYT and AYT, and correlation of BVP with PYT and AYT (B0.1).*

### Annual Genetic Gain

Annual genetic gain represents how much genetic gain have been obtained from each breeding cycle. Annual genetic gain for PS and GS was compared and the result for the five tested scenarios of PS and GS is presented in Table 1. Breeding programs that use genomic information has higher genetic annual genetic gain in all the tested scenarios than breeding program with phenotypic information only. For PS, annual genetic gain range from 0.035 to 0.049 while for GS the range was from 0.183 to 0.186 genetic standard deviation. Compared to PS, GS breeding scheme has tripled genetic gain. This increase in genetic gain when using GS was seen across the different levels of genetic correlations between traits. However, there was no significant differences of among the different levels of genetic correlations with in GS as well as PS.

### Genetic Variance per Cycle

**FIGURE 4:**
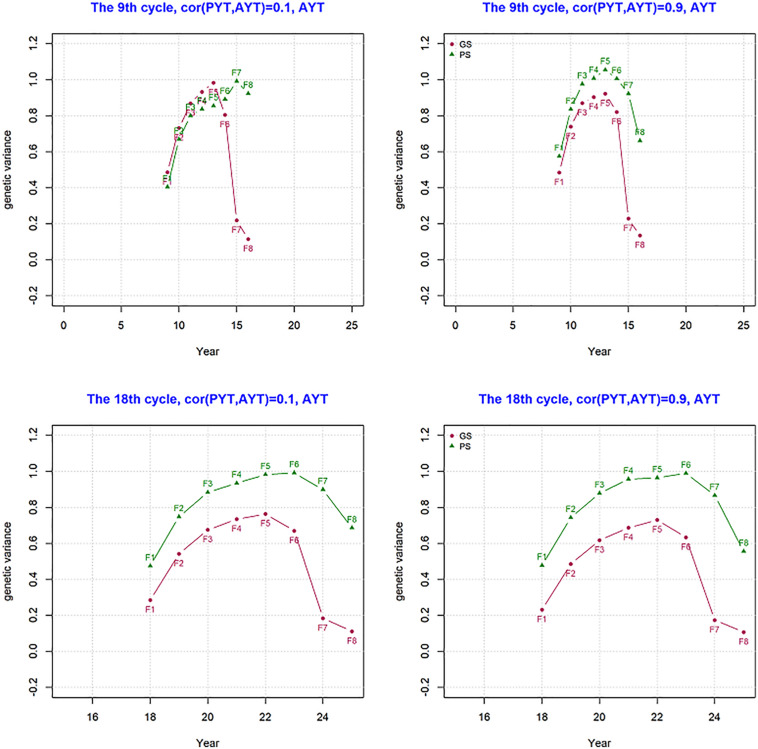
Genetic variance for AYT (advanced yield trial) in a singe breeding cycle from F1 to F8 at year 9 and 18 for genetic correlation of 0.1 and 0.9 between PYT and AYT.

Genetic variance per cycle was measured and this shows how much of the genetic variance created by crossing and recombination is reduced along the different stages of selection within a breeding cycle. Genetic variance for trait AYT was measured within a breeding cycle for PS and GS breeding schemes and the result is shown in Figure 4. The figure shows what happens in a single breeding cycle (F1 to F8) that started in the year 9 and 18 of the breeding program as an example for both PS and GS breeding schemes. In both year 9 and 18 of the breeding programs, an increase in genetic variance happened from F1 to F5 due to recombination. This trend is seen for both PS and GS breeding schemes. Genetic variance at F1 is 0.5 which is half of parent’s variance and this is expected since the parents are inbred and Mendelian sampling is 0. The decline in genetic variance after F5 comes as a result of selection. After selection only the best lines are kept to proceed to the next breeding stage. In GS, the change in variance after F5 shows a sharp decline due to selection when compared to PS due to more accurate predicted breeding values.

### Genetic Variance Across Breeding Cycles

**FIGURE 5:**
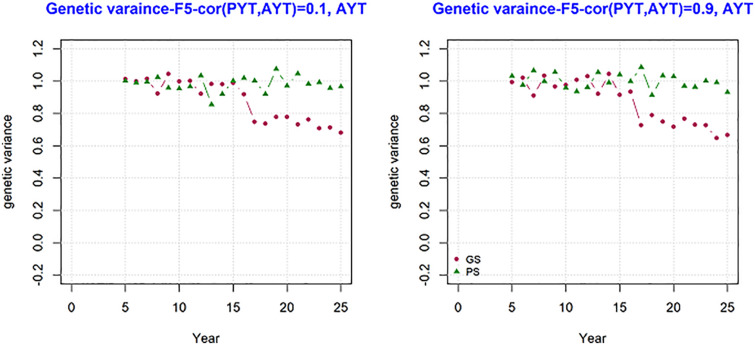
The change in genetic variance over the period of 25 years measured at generation F5 for AYT for GS (Genomic selection) and PS (Phenotypic selection) for genetic correlation of 0.1 and 0.9 between PYT (preliminary yield trial) and AYT (advance yield trial). The first 8 years for GS is burn-in and similar to PS.

The development of genetic variance over the period of 25 years was measured and the result is present in Figure 5. The figure shows mean genetic variance for the trait AYT for the genetic correlation 0.1 and 0.9 over the period of 25 breeding cycles for generation F5. The variance at F5 shows how much variance is available for subsequent selection. The change in genetic variance for AYT was different between genomic and phenotypic breeding programs. In both PS and GS variance is 1 at year 5, however the change in the level of genetic variance is higher for GS than PS in the subsequent period of selection. This shows the loss in genetic variance is higher for GS than PS. By the end of the breeding program, PS has genetic variance about 40% more than GS when the correlation was 0.9.

At the end of the breeding program, the change in genetic variance for both genomic and phenotypic selection for grain yield measured for AYT for F5, F6, F7, and F8 at the end of the breeding cycle (25th year) together with the corresponding breeding values is shown in Table 2. The table shows mean genetic gain for PS and GS and it is significantly different between PS and GS. By the end of the breeding program GS has produced about 300% more gentic gain than PS. The loss in genetic variance was higher in genomic selection than phenotypic selection in all teseted scenarios.

**TABLE 2:** Genetic gain and genetic variance for AYT for generation F5, F6, F7, and F8 at the end of the breeding program (year 25) for phenotypic (PS) and genomic selection (GS) of each scenario.

		**Genetic gain**		**Genetic variance**	
	
		**GS**	**PS**	**GS**	**GS**
**Scenario**	**Generation**	**mean(se)**	**mean(se)**	**mean(se)**	**mean(se)**
	F5	3.07(0.03)	0.74(0.07)	0.68(0.03)	0.97(0.04)
	F6	3.35(0.07)	0.61(0.05)	0.62(0.04)	0.96(0.05)
	F7	4.11(0.07)	0.84(0.06)	0.15(0.02)	0.94(0.07)
	F8	4.48(0.09)	1.00(0.18)	0.11(0.01)	0.69(0.14)
B0.1_Y0.3	F5	3.14(0.03)	0.74(0.06)	0.61(0.01)	0.96(0.03)
	F6	3.40(0.04)	0.84(0.10)	0.57(0.04)	1.01(0.05)
	F7	4.16(0.05)	1.04(0.10)	0.17(0.01)	0.99(0.05)
	F8	4.46(0.09)	1.05(0.08)	0.08(0.01)	0.77(0.16)
B0.1_Y0.5	F5	3.15(0.03)	0.87(0.06)	0.68(0.01)	0.98(0.06)
	F6	3.41(0.08)	0.96(0.09)	0.51(0.02)	0.95(0.05)
	F7	4.15(0.05)	1.15(0.09)	0.16(0.01)	0.96(0.10)
	F8	4.50(0.09)	1.28(0.10)	0.12(0.02)	0.82(0.17)
B0.1_Y0.7	F5	3.15(0.03)	0.89(0.07)	0.61(0.01)	0.93(0.02)
	F6	3.47(0.05)	0.88(0.05)	0.60(0.03)	1.01(0.04)
	F7	4.18(0.04)	1.10(0.10)	0.18(0.02)	0.88(0.08)
	F8	4.46(0.08)	1.30(0.12)	0.12(0.02)	0.75(0.14)
B0.1_Y0.9	F5	3.14(0.04)	1.03(0.08)	0.67(0.01)	0.93(0.04)
	F6	3.36(0.04)	1.08(0.07)	0.55(0.02)	0.97(0.03)
	F7	4.24(0.04)	1.44(0.10)	0.19(0.01)	0.99(0.07)
	F8	4.52(0.07)	1.19(0.15)	0.11(0.01)	0.56(0.09)

*The five levels of correlation (Y0.1, Y0.3, Y0.5, Y0.7, and Y0.9) between PYT and AYT, and correlation of BVP with PYT and AYT (B0.1).*

### Predicition Accuracy

Predicition accuracy of breeding value was computed as correlation between TBV and predicted breeding values. The mean predicition accuray of plot yield for PYT and AYT from year 9 to 25 for generation F6, F7, and F8 is presented in Table 3. In all the three generations GS had higher predicition accuracy than PS. For PS, accuracy was 0.269, 0.76, and 0.755 for F6, F7, and F8, respectively, while for GS the accuracy was 0.397 for F6, 0.992 for F7 and 0.966 F8. Among the generations, F7 and F8 has the highest accuracy for both PS and GS.

**TABLE 3:** The mean (se) accuracy of F6, F7, and F8 across the entire breeding cycle for cor(PYT,AYT) = 0.9 scenario.

	**Accuracy**	

**Generation**	**PS**	**GS**
F6	0.269(< 0.001)	0.397(< 0.001)
F7	0.763(< 0.001)	0.992(< 0.001)
F8	0.755(< 0.001)	0.966(< 0.001)

## Discussion

The current wheat breeding simulation study was done to test a hypothesis that genomic selection can increase genetic gain compared to phenotypic selection. For testing this hypothesis, a wheat breeding program was simulated for both genomic and phenotypic selection. The breeding program ran for 25 years. Within PS and GS breeding programs, different level of genetic correlation between PYT and AYT was tested. Our result confirmed the hypothesis that wheat-breeding program that used genomic information has tripled genetic gain compared to the conventional phenotypic selection.

### Genetic Gain

Genomic selection breeding schemes has outperformed phenotypic selection for genetic gain. The increase in genetic gain that was brought by adding genomic information in conventional wheat breeding program was 3-fold. The main advantage of using genomic selection was increasing selection accuracy which inturn increased genetic gain, which was cosistent with the finding of Gaynor et al. (2017). Our result have shown that selection accuracy was higher in GS than PS. The use of genomic information to select parental lines has been shown to contribute to an increase in genetic gain through enhancing selection accuracy (Gaynor et al., 2017). In our simulation, lines that were selected based on their breeding values were stored to be used potentially as parents in the preceding cycles. However, parents were selected randomly from the stored line for the subsequent cycle. In crop breeding, selection of parents are one of the most important steps as it determines the direction of change in the genetic improvement (to bring genetic progress) (Akdemir et al., 2019).

In the current study, the increase in genetic gain was about 300% more when genomic information was used for selection. Previous study (Gaynor et al., 2017) has reported about 1.21 times more genetic gain when using genomic information in wheat breeding program. However, in our study GS had produced more genetic gain than reported by Gaynor et al. (2017). This huge difference was because genetic gain reported for phenotypic selection in the current study was very much lower than what was reported by Gaynor et al. (2017) while for GS we did not observe huge difference in reported genetic gain with their studies.

Rate of genetic gain is used to compare an outcome from different breeding schemes that will help in designing a new breeding program (Rutkoski, 2019). Our study showed that the increase in genetic gain that came by adding genomic information at the preliminary selection stage could help a wheat breeder for practical decision making to switch to GS breeding program. This study can provide a guideline on how to apply genomic selection starting from the preliminary yield trial. Preliminary yield trial (PYT) is un-replicated and limited amount of seed is available for each selection candidate. However, this selection step strongly influences the subsequent advanced yield trial (AYT), which are commonly tested in multiple locations (Poland et al., 2018). In addition, GS at PYT allows for early elite parental selection for the next breeding cycles. In the current study, we assumed no genotype by environmental interactions in the advanced yield trial and this might overestimate the advantage of GS over PS.

### Additive Genetic Variance

Selection causes changes in variances, allele frequencies, and LD relationships between markers and QTL (Bulmer, 1971; Muir, 2007). In our simulation study, the change in genetic varaince over time had a different trend for genomic selection and phenotypic selection. The loss in genetic variance over time was higher for genomic selection than for phenotypic selection. A similar result where genomic selection decreases genetic variance in wheat breeding prgram more than phenotypic selection was reported by Gaynor et al. (2017). By the end of the breeding program (year 25), about 66 % of genetic varaince was available for GS when the correlation between PYT and AYT is 0.9 while for PS it was about 90%. However, Gaynor et al. (2017) showed that about 33% genetic varaince was available for conventional genomic selection program. This difference could be related to the number of years of the breeding program, the current study is 25 years while in Gaynor et al. (2017) breeding programs runs for 40 years with 20 years of burn-in period. In addition, the number of lines for AYT is higher (150) in the current study while in Gaynor et al. (2017) which was 50 lines.

The change in genetic variance within a breeding cycle at the eary stage (year 9) and later stage (year 18) of the breeding program clearly showed differences in genetic varaince between GS and PS. Genetic varaince within a breeding cycle is produced through recombination until F5, after this stage varaince starts to decline faster because of selection. At the early stage of the breeding program (year 9), more genetic varaince was available for GS, however, as selection continues variance starts to decline for as seen in year 18. It also showed that the loss in genetic varaince is accelerated in GS than PS.

In our study, the change in genetic variance depending on the genetic correlation between PYT and AYT was not significantly different from each other. Additive genetic variance plays an important role in predicting the change in the population mean due to selection (Bernardo, 2010). That means if there are more variance present in a population, more genetic improvement in a population is possible. The current simulation assumes closed breeding program, which might exacerbate the loss of genetic variance. In real breeding program, breeders exchange breeding materials that will help introduce genetic variance to the on-going breeding program. Thus introducing breeding materials from outside can help in reducing the faster decline of genetic variance. Furthermore, the increased rate of decline in genetic variance when applying genomic selection calls for more measures to ensure sufficient genetic variance in future generations of parents. Including methods of optimum contribution selection should be investigated as means of controlling the loss of genetic variance in future generations of parents (Cowling et al., 2017; De Beukelaer et al., 2017; Gorjanc et al., 2018).

This study has shown considerable advantage of genomic selection over conventional phenotypic selection for winter wheat-breeding programs by drastically improving genetic gain. Generating more genetic gain implies additional revenue, although generating additional revenues may add additional costs such as genotyping. Accuracy in GS, dependes on the size of the training population which are required to be genotyped which may add extra cost. Thus, it is important to determine the optimum training population size without compromizing the selection accuracy. However, genotyping costs are decreasing and this makes GS a more efficient tool to bring genetic improvement in cereal breeding than PS (Robertsen et al., 2019).

This simulation was done following commercial wheat breeding schemes, including the number of families and single plants meaning the result easily can be translated into practical wheat-breeding programs. Genomic selection has increased response to selection with better estimates of breeding values and more accurate selection of future parents. We believe this study will help in desicion making process for a wheat breeder to switch to genomic selection. In addition to implementing genomic selection to increase genetic gain, accelerating the breeding cycle through speed breeding is also a promising approach to increase genetic gain that needs further investigations.

## Conclusion

The current study shows that incorporation genomic information in conventional wheat-breeding program can increase genetic gain. Besides, using genomic selection for a conventional wheat-breeding program requires a minimal change in an already existing breeding program. The increase in genetic gain in GS was mainly due to an increase in selection accuracy. Individuals selected based on their GEBVs were stored to be used as a parents. Genetic variance was reduced higher in GS than in PS, indicating it is necessary to incorporated optimum contribution selection in order to conserve genetic variation in the breeding program.

## Data Availability Statement

The raw data supporting the conclusions of this article will be made available by the authors, without undue reservation.

## Author Contributions

BBT performed the analyses and prepared the manuscript. All authors helped in designing breeding scenarios, interpreting the results, and reviewing the manuscript. All authors read and approved the final manuscript.

## Conflict of Interest

JA was employed by company Nordic seed. The remaining authors declare that the research was conducted in the absence of any commercial or financial relationships that could be construed as a potential conflict of interest.
